# HIV status and treatment influence on fertility desires among women newly becoming eligible for antiretroviral therapy in western Kenya: insights from a qualitative study

**DOI:** 10.1186/s12978-017-0355-9

**Published:** 2017-08-08

**Authors:** James Ayieko, Angeline Ti, Jill Hagey, Eliud Akama, Elizabeth A Bukusi, Craig R Cohen, Rena C Patel

**Affiliations:** 1 Centre for Microbiology Research, Kenya Medical Research Institute, Kisumu, Kenya; 2 Department of Obstetrics, Gynecology and Reproductive Sciences, University of California, San Francisco, USA; 3 Department of Family and Community Medicine, University of California, San Francisco, USA; 4 School of Medicine, University of California, San Francisco, USA; 5 Departments of Obstetrics and Gynecology and Global Health, University of Washington, Seattle, USA; 6 Department of Medicine, University of Washington, Seattle, USA

**Keywords:** HIV-infected women, ART-naïve, Fertility desires, Kenya

## Abstract

**Background:**

Factors influencing fertility desires among HIV-infected individuals remain poorly understood. With new recommendations for universal HIV treatment and increasing antiretroviral therapy (ART) access, we sought to evaluate how access to early ART influences fertility desires among HIV-infected ART-naïve women.

**Methods:**

Semi-structured in-depth interviews were conducted with a select subgroup of 20 HIV-infected ART-naïve women attending one of 13 HIV facilities in western Kenya between July and August 2014 who would soon newly become eligible to initiate ART based on the latest national policy recommendations. The interviews covered four major themes: 1) definitions of family and children’s role in community; 2) personal, interpersonal, institutional, and societal factors influencing fertility desires; 3) influence of HIV-positive status on fertility desires; and 4) influence of future ART initiation on fertility desires. An iterative process of reading transcripts, applying inductive codes, and comparing and contrasting codes was used to identify convergent and divergent themes.

**Results:**

The women indicated their HIV-positive status did influence—largely negatively—their fertility desires. Furthermore, initiating ART and anticipating improved health status did not necessarily translate to increased fertility desires. Instead, individual factors, such as age, parity, current health status, financial resources and number of surviving or HIV-infected children, played a crucial role in decisions about future fertility. In addition, societal influences, such as community norms and health providers’ expectations of their fertility desires, played an equally important role in determining fertility desires.

**Conclusions:**

Initiating ART may not be the leading factor influencing fertility desires among previously ART-naïve HIV-infected women. Instead, individual and societal factors appear to be the major determinants of fertility desires among these women.

## Plain English summary

As access to life-saving treatment for HIV with antiretroviral therapy becomes universally available across the world, it is important to understand how treatment for HIV may change HIV-infected women’s desires to have more children. We conducted interviews with HIV-infected women in Kenya who had previously never been on HIV treatment to help us understand what their desires for having more children are, and how the possibility of starting HIV treatment soon may change their desires. The results of our study suggest that HIV-positive status greatly affects these women’s desires for having more children, and mostly in a negative manner in discouraging them from having more children. The women thought that starting HIV treatment soon would improve their health status but not necessarily increase their desire to have more children. Instead, we found that a separate set of individual and societal factors comes into play in influencing their desires for having more children. Individual factors, such as their current age, the number of children they already have, and how many of those children are still alive or infected with HIV, more directly influence their desires for having more children. Societal factors, such as community norms for motherhood and health providers’ expectations of their desires, also play an equally important role in influencing their desires for having more children. The findings of this study are crucial for health care providers and HIV treatment programs to understand in order to meet their HIV-infected women’s reproductive health needs.

## Background

The latest World Health Organization (WHO) guidelines on HIV treatment now recommend initiating antiretroviral therapy (ART) in all individuals living with HIV, regardless of CD4 cell count or disease severity [1]. This recommendation is informed by a growing body of evidence that highlights the benefits of early ART initiation [2–4], including reduced mortality [5] and morbidity among HIV-infected individuals [1, 6]. Expanding access to ART in resource-limited settings over the last two decades has transformed the disease to a manageable chronic illness [7, 8] and allows HIV-infected individuals an improved overall quality of life [9] in these settings. Additionally, a study conducted in Uganda demonstrates that HIV-infected individuals now have similar life expectancy to the general population [10].

With increased life expectancy and improved quality of life [5, 6, 11], HIV-infected individuals now face decisions and considerations that they previously did not encounter. One such consideration is their fertility desires [12]. Few studies have evaluated the impact of HIV infection on fertility desires and pregnancy rates among HIV-infected women, though some evidence supports lower fertility desires among HIV-infected women compared to their HIV-uninfected peers [13, 14]. However, this gap in fertility desires appears to diminish with improvements in the health status of HIV-infected women [15, 16]. Additionally, only one study has examined ART’s influence on fertility desires [17], and to our knowledge, none have examined the influence of earlier ART initiation at higher CD4 cell counts, where the anticipated benefit of improvement in health status is lower. Up until July 2014, the Kenyan HIV treatment guidelines recommended initiating ART only at CD4 cell counts <350 cells/mm^3^ or with WHO clinical disease stage of III or IV [18]. Thereafter, the Kenyan guidelines included ART initiation for individuals with CD4 cells counts between 350 and 500 cells/mm^3^ [19], and since July 2016, has recommended ART initiation for all HIV-infected individuals regardless of CD4 cell counts [20]. As access to ART becomes universal, it is imperative to understand the fertility desires among HIV-infected individuals so that HIV treatment programs can meet their patients’ reproductive health needs.

Given the importance of understanding factors that influence fertility desires among HIV-positive women and how access to earlier ART modulates their fertility desires, we sought to examine how earlier ART initiation may influence future fertility desires. We conducted this qualitative study among a group of HIV-infected but ART-naïve women of reproductive age from western Kenya who meet the new country guidelines for initiating ART.

## Methods

We conducted in-depth interviews with 20 HIV-positive ART-naïve women between July and August 2014 attending one of 13 HIV facilities in western Kenya. These facilities are supported by Family AIDS Care & Education Services (FACES), a collaboration between the University of California, San Francisco and the Kenya Medical Research Institute [21]. These facilities were located in Homabay, Kisumu, and Migori counties and ranged from County hospitals to health centers; they have been described in greater detail elsewhere [22]. We purposively selected facilities in each county/sub-county based on its representativeness of the region, accessibility, and level of health service provision. At each facility, we used a convenience sample to recruit 1–3 women to interview.

Study staff approached the nurse in-charge at each facility at the beginning of the clinic day and requested in-person referrals of 1–3 women attending the facility that day who were 18–45 years of age, non-sterilized, HIV-positive, ART-naïve, and had a CD4 cell count between 350 and 500 cells/mm^3^ within the last six months. Therefore, we sampled women who were ART-naïve but would be initiating ART in the coming weeks under the new Kenyan guidelines. Since the policy change in CD4 count threshold for ART initiation had changed in Kenya the same month of our study initiation, study participants were not aware of this newly changed treatment policy at the time of conducting the interviews. The study staff approached the potential participants and assessed interest for study participation.

The interviews were conducted in DhoLuo by a trained, female interviewer and digitally recorded. The interviewer confirmed study eligibility, obtained written informed consent, collected basic demographic information, and then started the interview. The interviewer used a semi-structured interview guide to prompt discussions on the following themes: 1) definitions of family and children’s role in family/community; 2) personal, interpersonal, institutional, and societal factors influencing fertility desires; 23) influence of HIV-positive status on fertility desires; and 34) influence of future ART initiation on fertility desires. The interviewer transcribed the initial two interviews in DhoLuo and then translated these into English. Another member of the study staff verified the accuracy of the English translations against the audio file and DhoLuo transcripts and resolved any discrepancies. Thereafter, the interviewer translated the interview audio files directly into English. Transcripts were coded manually using a word processor, and the coded text was further organized into themes and subthemes in separate documents. Transcripts from the interviews were iteratively analyzed using inductive content analysis [23]. An initial codebook was developed from the interview guide, which was further refined with discussion and consensus as the initial transcripts were coded. The first three transcripts were double coded by two members of the study team (A.T. and R.C.P.) and differences in coding were resolved through discussion until consensus was reached. After all the data were coded, the investigators used an iterative process of reading transcripts, comparing and contrasting codes, and identifying convergent and divergent themes.

## Results

### Participant characteristics

All 20 participants were ART-naïve women with a median age of 30 years. Thirteen were currently married, 12 completed primary education, and 16 lived in rural areas. The majority (17) of the women were currently using a contraceptive method and had a median of four living children at the time of the study (Table 1).

**Table 1:** Baseline characteristics of participants

Variable	HIV-infected and ineligible for ART (*n* = 20)
Sociodemographic	
Age (years), median	30 (range 20–45)
Education status	
Did not complete primary school	7
Completed primary school	12
Completed secondary school or more	1
Marital status	
Currently married	13
Widowed/separated	7
Habitation	
Urban	4
Rural	16
Household size inclusive of participant	4 (range 1–11)
Reproductive health-related	
Total number of pregnancies	4 (range 0–8)
Pregnancies resulting in live births	4 (range 0–8)
Number of living children	4 (range 0–8)
Prior contraceptive method use^1^	
Implants, e.g. Norplant	2
Injectables, e.g. DMPA	13
Oral pills	4
Condoms	3
None	1
Current contraceptive method use	
Implants, e.g. Norplant	3
Injectables, e.g. DMPA	9
Oral pills	0
Condoms	5
None	3
HIV-related	
Duration of known HIV positive status (years), median	1.1 (range 0.06–11.7)
Most recent CD4 count cells/mm^3^, median	421 (range 350–500)

^1^Adds to more than 100% as multiple methods were reported by some women

### Role of children in the community

According to participants, children have several important roles they fulfill for families, and are thus highly valued by the women and their communities. First, children are valued for the help they offer in performing domestic chores, such as cleaning, cooking, herding animals, and running errands. Second, these children offered emotional value to the women, by offering companionship to the women as well as making the women feel more secure around the house. One woman stressed that children strengthen the relationship between spouses, terming children as “the greatest bond between a husband and a wife” (*26 year-old, three living children, widowed from rural area*)*.* Third, women stressed that having children was a future investment, implying that educating their child helps ensure that the child will secure a better future for her/himself, and be in a better position to help care for their family as they grow old. Lastly, the women indicated that children are associated with higher social status and more respect in the community; not having children is highly stigmatizing. These women felt that their communities expect women to bear children once they are married for the purpose of propagating the family lineage or as replacement for relatives who had passed away.

“The role of children in the village is good, because these children are the ones who uplift the village. They can be educated to become engineers, ministers, and the likes. You also find that the communities with children have a lot of development.” *37 year-old, three living children, married from rural area.*

“Children bring joy in the house when their father is not around. They also offer me protection when their father is not around.” *29 year-old, two living children, married from urban area.*

“The community looks down upon those who do not have children. If it’s my brother who has such a wife who doesn’t give birth, he will be rebuked and not regarded as somebody in the community. Someone who has a child is being called ‘his daughter/son’s father’ and that gives him much respect in the family. Disrespect comes when you don’t have the child, but if you have children then you have so much respect in the community (talks to the baby who is crying on her lap).” *28 year-old, three living children, married from urban area.*

### Fertility intentions differ by HIV status

#### Community expectations of fertility differ by HIV status

Nearly all women interviewed indicated that community expectations of fertility differ by HIV infection status. Generally, they felt that their communities expect and support HIV-uninfected women to have children unequivocally. In some instances, the community even pressures a woman to have children, as long as the woman is married, so as to fulfill societal expectations of fertility.

For HIV-infected women, however, our participants reported varying perceptions of community expectations of their fertility. Most women perceived the community to discourage fertility among HIV-infected women, for three major reasons. First, they felt that their communities fear that an HIV-infected woman might die soon, and, therefore, burden the community with the care of their now orphan. Second, they felt their communities are concerned that an HIV-infected woman can transmit the infection to her infant or uninfected spouse while pregnant or attempting conception. Finally, they perceived their community members feared that childbearing might worsen the woman’s health status. Nearly all the women indicated that these community concerns were only communicated to them indirectly.

“The community would not really desire for HIV-infected women to have children as they get worried about you dying and leaving the children under their care. They also see it as a way of spreading the infection because whenever one needs to conceive they do not have protected sex. This makes people to talk a lot behind your back as they cannot face you with whatever they have to say.” *25 year-old, two living children, married from urban area.*

“A woman with a positive status is not expected to have more children because it will be considered that the born child would be positive and the woman will die faster, while an HIV-negative woman will be viewed normally because she has nothing to be talked about.” *43 year-old, five living children, married from rural area.*

A few women, however, felt that community expectations of their fertility did not differ from uninfected women, largely because their community members were not aware of their positive HIV status. One participant indicated that she perceived minimal community stigma against HIV-infected women, as she considers HIV infection equivalent to other illnesses, such as malaria.

“Stigma around HIV used to happen earlier when we were still girls, but these days I don’t see it happening. These days we see it (HIV) as just malaria, which needs to be treated.” *28 year-old, three living children, married from urban area.*

#### Health provider attitudes towards fertility differ by HIV status

Nearly all women indicated that they perceived health provider attitudes towards fertility also differing by HIV status. They reported that HIV-infected women are routinely discouraged from conceiving until their immunity is “acceptable”.

“Whenever the healthcare providers know that you are HIV positive they advise for a CD4 count, nutrition status, haemoglobin, and weight confirmations. A check is also done for some diseases like diabetes. After these numerous tests, one is given the go ahead to conceive.” *25 year-old, two living children, married from urban area.*

“The healthcare providers must first check the CD4 level to verify if she is fit to give birth. Then once she gives birth, some protective measures are taken for the sake of the infant.” *27 year-old, two living children, married from rural area.*

Consequently, these women felt that health care providers more frequently recommended contraceptives to HIV-infected women as compared to uninfected women. They perceived providers considering contraception, alongside ART, as a tool in helping to improve the infected women’s immunity to a level where they can conceive safely. Generally, the women agreed that delaying child bearing by using effective contraception, provides them an opportunity to help improve their immunity in the interim; they perceived pregnancy and childbirth as processes that would invariably lower their immunity.

“They (health care workers) will only advise her (an HIV-infected woman) to use contraceptives, such as condoms, and also how to protect the unborn child from being infected. There is a difference in how they (healthcare workers) react to HIV-infected women compared to HIV-negative women…an HIV-infected woman’s life depends much on health care providers’ advice, as compared to an HIV-negative woman who is only advised on how to protect herself from getting infected with HIV.” *26 year-old, three living children, widowed from rural area.*

#### Women’s fertility intentions differ by HIV status

These HIV-infected women perceived their fertility intentions to differ from those of HIV-uninfected women. They indicated that HIV-infected women have several HIV-specific factors to consider before conception, which can be grouped into the following subthemes: 1) health consequences of pregnancy for HIV-infected women; 2) financial stress of pregnancy in HIV-infected women; and 3) emotional and financial burden of already having HIV-infected children.

In contrast to the above HIV-specific factors that HIV-infected women have to consider when considering conception, the participants felt strongly that because HIV-uninfected women are healthier than infected women, they have fewer health-related worries.

“HIV-positive women often have many health problems and that is why they think that when they deliver a baby, the baby will be often affected by health problems too. However, an HIV-negative woman views themselves as without any health problems.” *25 year-old, two living children, married from urban area.*

“They have to consider that they are on medication which only aids to prolong their lives so having more children will make their lives difficult. HIV-positive women have a lot of considerations to make when wanting children as compared HIV-negative women who can just have a child whenever she feels like. (Pauses.) HIV-positive women have the thought that they might give birth to infected children or their CD4s might be low hence causing more complications to them.” *27 year-old, two living children, married from rural area.*

Below we discuss in detail the three major subthemes of HIV-related factors infected women have to consider before conception.

##### Health consequences of pregnancy differ for HIV-infected women

First, most women based their fertility intentions on their perceived immune status as the leading factor to consider for any health consequences of a pregnancy. Specifically, these women indicated using their CD4 cell count as a marker of immunity, with a firm belief that conception at low immunity would lead to further deterioration of their health.

“I wanted to have four children before I knew my HIV status, but now that I know my status, even two children are just enough for me. (Laughing) Two are enough for me. I don’t know God’s plan, but I hope God will someday add me one more (child) so that they become three. Right now, my haemoglobin is low and my CD4 is low too. I feel that sometimes I can add children, but then I may die leaving them when they are still young.” *30 year-old, two living children, married from rural area.*

“The things that come to mind when deciding on whether or not to have more children is one’s life. One thinks about how their life will be when they conceive. You wonder whether your immunity will be boosted or lowered. People think that once you conceive and the infections come up, you are not able to eat well and this really reduces your CD4 counts.” *25 year-old, two living children, married from rural area.*

Second, many women feared that pregnancy-related complications, such as post-partum bleeding, were more likely to occur in HIV-infected as opposed to uninfected women, which would further deteriorate their health.

“I know I can get a child…However, one has to consider how your life will be. One might loose a lot of blood during delivery, and this would be a setback to one’s health.” *25 year-old, two living children, married from rural area.*

Third, a few women also pointed out that being in a sero-discordant relationship is itself a deterrent to having more children due to the fear of HIV transmission to their uninfected partner during unprotected sex.

“HIV-positive women think of the status of their spouses, like in my case, I am a widow and I am HIV-positive. Sometimes the man you have is HIV-negative, so you think about whether you will transmit the infection to him or not.” *30 year-old, two living children, widowed from rural area.*

“This is the difference in what HIV-negative and positive women must consider when wanting to have children, when an HIV-negative woman has sexual intercourse with her husband she can’t think of anything else. But if you know you are HIV-positive and you want to get pregnant, it’s a must that you avoid condom use. So you must look for information so that you don’t hurt the other person.” *27 year-old, no living children, married from rural area.*

##### Financial stress of pregnancy differ for HIV-infected women

Some women pointed out that pregnancy is more financially demanding for an HIV-infected versus uninfected woman, another HIV-specific factors influencing fertility desires. One woman reported that there were additional costs associated with closer medical follow-up and being on ART when pregnant, including a perceived “special diet” that ART requires.

“Whenever one conceives, it is a burden as one is expected to eat very well, especially when they are HIV-positive…A person who is HIV-positive is expected to eat well, especially foods like vegetables. Then another problem that expectant women face is that they become choosy on different foods. This makes it tricky! This is a very big challenge for HIV-positive expectant women.” *25 year-old, two living children, married from urban area.*

##### Emotional and financial burden of already having HIV-infected children

Some women also took into account the HIV status of their existing children and the emotional and financial burden of the children’s HIV care in determining their fertility intentions. These women reported feeling discouraged by already having an HIV-infected child, and would, therefore, not desire to have future children who could also possibly be HIV-infected. Some women also stressed that having an ill HIV-infected child was already a large burden for them.

“My child’s HIV-positive status really preoccupies my mind. Recently the child has been sickly, so this makes me not to want more children. Let me just take care of the ones I already have.” *27 year-old, two living children, married from rural area.*

“After my child contracted HIV, I just felt that I should not give birth again. This experience discouraged me from giving birth as I just felt that even if I gave birth to another child, the child could also contract the virus. The reason why I do not want more children now is because, (laughing) I feel those children that I already have are enough.” *30 year-old, three living children, married from rural area.*

### ART’s potential influence on fertility intentions

The participants’ opinions on how initiating ART would affect their fertility intentions varied widely, with some women anticipating no change and others perceiving positive or negative influences. Some women felt that ART would improve their health or immunity and would, therefore, motivate them to pursue having more children.

“When I start using ART, if I use them well then my CD4 count will go up. Then I can wish for God to add me one more child. That’s my wish, so that they are three, that is if I am responding well to the drugs.” *30 year-old, two living children, married from rural area.*

Some women felt their fertility desires would not change with initiation of ART because they perceived their lifespan being shortened by HIV infection, despite future ART use, and continued to not want to orphan a future child. A few women, however, did not think that initiating ART would change their fertility desires, largely because they were content with the children they already had.

“Starting ART won’t change how I think about having children; I will still not want more children. I am content with what children I already have.” *35 year-old, four living children, separated/divorced from rural area.*

Lastly, a minority of the women felt that initiation of ART would negatively impact their fertility desires. They considered the use of ART as an additional burden to their life, and, thus, felt ART use would diminish their fertility desires.

“Starting ART will change how you think about having a child. This is because it will be tiresome to take care of the child as you also take care of yourself. It will also affect your business and other activities since it (using ART) will need much concentration.” *22 year-old, two living children, married from rural area.*

Generally, other factors, such as increasing parity and age, appeared to more strongly and negatively influence the women’s fertility desires. For example, women who already had children before they knew their positive HIV status reported less desire to have more children in the future, with a majority indicating that they felt content with the number of children they already had. However, women who did not have any children expressed stronger desires to have children despite their positive HIV status.

“Before I knew that my status had changed, I had already decided not to have any more children. The five I have are just enough for me to take care of.” *43 year-old, five living children, married from rural area.*

*“*I see myself not giving birth anymore…. I wish to continue with family planning methods so that I don’t get pregnant again.” *41 year-old, eight living children from rural area.*

“(Sighs) I don’t see how it (my personal health status) relates to the desire to give birth…I just wish…I still have the desire (to give birth).” *32 year-old, no living child, widowed from rural area.*

## Discussion

This qualitative study demonstrates a spectrum of beliefs on how HIV status and access to earlier ART may influence future fertility desires. The women indicated that their HIV positive status does influence—largely negatively—their fertility desires confirming the finding of other studies [15, 17, 24], though it was less clear if these desires would change after initiation of ART. Ultimately, the women indicated that children are highly valued by them and their communities; social norms, community expectations, and sociodemographic factors, such as their age and number of living children, appeared to have the strongest influence on their fertility desires.

That fertility desires differ by HIV status, and that HIV-infected women are discouraged from future pregnancies is not surprising. Maternal morbidity and mortality is a significant consideration for all women considering a pregnancy. HIV-infected women have to consider additional HIV-specific factors, such as the risk of perinatal or sexual transmission of HIV, their current health status, social expectations, health care provider’s recommendations, and the burden of raising a possibly infected child alongside caring for oneself and her surviving children [17, 25–30].

While a positive HIV status has a negative influence on fertility desires, our data demonstrates it is less clear if fertility desires will change with earlier initiation of ART, while a woman is relatively healthy. Our analysis reveals that HIV-infected ART- naïve women are largely aware that initiation of ART facilitates improved health status and life expectancy, providing them the opportunity to lead a “normal” life and pursue their goals and desires. Yet, for some women, initiating ART and anticipating improved health status does not necessarily translate to increased fertility desires. A few cross sectional surveys have evaluated fertility intentions among HIV-infected women on or off ART, and found no association between ART use, regardless of duration of ART use, and desire to have future children [26, 31, 32]. However, none of these studies further distinguish fertility desires among women with higher CD4 counts and good health status; it is possible that actual, instead of anticipated, fertility intentions might change after ART initiation among these women with higher CD4 cell counts, for example, due to quicker recovery of health and normalization of life.

Ultimately, these women may have additional, stronger influences on their fertility desires than the anticipated quality of life improvements with ART initiation. For instance, age, parity, and educational status strongly influence fertility [17, 24]. Our data supports the findings that women who already have children are less likely to desire additional children, while younger women without any children express a strong desire to have children [14, 24, 33].

What is interesting, however, is how large of an influence community expectations and beliefs had on these women’s fertility desires. The women found themselves navigating opposing perceived community expectations of their fertility. The women made it clear that, on one hand, children are highly valued by them and their communities. Having children is closely linked with higher social status and anticipated future economic returns, placing a high value on parenthood, especially motherhood, a finding well-demonstrated in settings both within [25] and outside of the context of HIV [29, 34–36]. On the other hand, these women perceived community expectations for HIV-infected women to not have future children, generally out of concern for future orphan-hood for existing children, and the community’s subsequent burden of care, or potential HIV transmission to infant or partner [17].

Nonetheless, our data highlights a few HIV-specific factors that may more strongly influence these women’s fertility desires than initiation of ART. Some women in our study considered raising a child an additional burden to caring for themselves, which was a priority for them. In addition, women with perinatally-infected children seemed less likely to desire additional children, largely due to the fear of having additional infected children [37]. Initiation of effective ART would, of course, lower the risk of transmitting future children; however, the women in our study did not seem to appreciate this future possibility. In general, these women should be better supported to realize their reproductive health goals, whether that is using safer conception options, including life-long ART, to conceive or effective contraception to prevent pregnancies [38]. Practically this requires HIV treatment programs to integrate reproductive health services into their mainstream HIV care and systematically and proactively inquire about HIV-infected individuals’ fertility desires, so that the appropriate services can be provided to those individuals.

Despite sampling from across a large region and saturation of themes, our study has several limitations. First, we only conducted one-time interviews which does not offer insight into changing fertility desires over time. Second, social desirability bias to underreport their fertility desires from fear of perceived negative approval from society [17] may have affected our study. To minimize such potential bias, the interviewer strived to ask questions in a balanced fashion and use non-judgmental body language. Finally, though we sampled women attending HIV facilities from diverse regions of western Kenya, there might be limits to the transferability of our findings.

## Conclusion

Our findings suggest that initiating ART among relatively healthy HIV-infected women previously not eligible for ART may not necessarily change their fertility desires. While a few HIV-specific factors, such as perceived immune suppression from pregnancy or already having perinatally-infected children, may influence fertility desires, it is individual and societal factors, such as social norms and community expectations, that appear to be the major determinants of fertility desires among ART-naïve HIV-infected women. Ultimately, HIV-infected women should be better supported to realize their reproductive health goals, through integration of such services into HIV care, systematic inquiry of fertility desires, and lastly the use of safer conception options, including ART, or effective contraception for pregnancy prevention.

## Abbreviations

ART: Anti-Retroviral Therapy
DMPA: Depo-Medroxyprogesterone Acetate
FACES: Family AIDS Care & Education Services
HIV: Human Immunodeficiency Virus
WHO: World Health Organisation

## References

[1] WHO (2015). Guideline on when to start antiretroviral therapy and on pre-exposure prophylaxis for HIV.

[2] The TEMPRANO ANRS 12136 Study Group (2015). A trial of early antiretrovirals and isoniazid preventive therapy in Africa. N Engl J Med.

[3] The INSIGHT START Study Group (2015). Initiation of antiretroviral therapy in early asymptomatic HIV infection. N Engl J Med.

[4] Cohen M, Chen Y, McCauley M, Gamble T, Hosseinipour M, Kumarasamy N (2015). Final results of the HPTN 052 randomized controlled trial: antiretroviral therapy prevents HIV transmission. J Int AIDS Soc.

[5] Hogg RS, O’Shaughnessy MV, Gataric N (1997). Decline in deaths from AIDS due to new antiretrovirals. Lancet.

[6] Palella FJ, Delaney KM, Moorman AC (1998). Declining morbidity and mortality among patients with advanced human immunodeficiency virus infection. HIV outpatient study investigators. N Engl J Med.

[7] WHO (2013). Global update on HIV treatment: results, impact and opportunities: WHO report in partnership with UNICEF and UNAIDS.

[8] Patel KK, Patel AK (2006). Future implications: compliance and failure with antiretroviral therapy. J Post Grad Med.

[9] Mwesigire DM, Wu AW, Martin F, Katamba A, Seeley J (2015). Quality of life in patients treated with first-line antiretroviral therapy containing nevirapine or efavirenz in Uganda: a prospective non-randomized study. BMC Health Serv Res.

[10] Mills EJ, Bakanda C, Birungi J, Chan K, Ford N, Cooper CL (2011). Life expectancy of persons receiving combination antiretroviral therapy in low-income countries: a cohort analysis from Uganda. Ann Intern Med.

[11] Hogg RS, Heath KV, Yip B (1998). Improved survival among HIV-infected individuals following initiation of antiretroviral therapy. JAMA.

[12] Nobrega AA, Oliveira F, Galvao MT, Mota RS, Barbosa RM (2007). Desire for a child among women living with HIV/AIDS in northeast Brazil. AIDS Patient Care & STDs.

[13] Hoffman IF, Martinson FE, Powers KA, Chilongozi DA, Msiska ED, Kachipapa EI (2008). The year-long effect of HIV-positive test results on pregnancy intentions, contraceptive use, and pregnancy incidence among Malawian women. J Acquir Immune Defic Syndr.

[14] Heys J, Kipp W, Jhangri GS, Alibhai A, Rubaale T (2009). Fertility desires and infection with the HIV: results from a survey in rural Uganda. AIDS.

[15] Cooper D, Moodley J, Zweigenthal V, Bekker LG, Shah I, Myer L (2009). Fertility intentions and reproductive health care needs of people living with HIV in cape town, South Africa: implications for integrating re- productive health and HIV care services. AIDS Behav.

[16] Maier M, Andia I, Emenyonu N, Guzman D, Kaida A, Pepper L (2009). Antiretroviral therapy is associated with increased fertility desire, but not pregnancy or live birth, among HIV+ women in an early HIV treatment program in rural Uganda. AIDS Behav.

[17] Wekesa E, Coast E (2014). Fertility desires among men and women living with HIV/AIDS in Nairobi slums: a mixed methods study. PLoS One.

[18] NASCOP (2011). Guidelines for antiretroviral therapy in Kenya.

[19] NASCOP (2014). Guidelines on use of antiretroviral drugs for treating and preventing HIV infection.

[20] NASCOP (2016). Guidelines on use of antiretroviral drugs for treating and preventing HIV infection.

[21] Kulzer JL, Penner JA, Marima R, Oyaro P, Oyanga AO, Shade SB (2012). Family model of HIV care and treatment: a retrospective study in Kenya. J Int AIDS Soc.

[22] Hagey JM, Akama E, Ayieko J, Bukusi EA, Cohen CR, Patel RC (2015). Barriers and facilitators adolescent females living with HIV face in accessing contraceptive services: a qualitative assessment of providers’ perceptions in western Kenya. J Int AIDS Soc.

[23] Elo S, Kyngäs H (2008). The qualitative content analysis process. J Adv Nurs.

[24] Myer L, Morroni C, Rebe K (2007). Prevalence and determinants of fertility intentions of HIV-infected women and men receiving antiretroviral therapy in South Africa. AIDS Patient Care & STDs.

[25] Nattabi B, Li J, Thompson S, Orach C, Earnest J (2009). A systematic review of factors influencing fertility desires and intentions among people living with HIV/AIDS: implications for policy and service delivery. AIDS Behav.

[26] Kaida A, Laher F, Strathdee SA, Janssen PA, Money D (2011). Childbearing intentions of HIV-positive women of reproductive age in Soweto, South Africa: the influence of expanding access to HAART in an HIV Hyperendemic setting. Am J Public Health.

[27] Lam PK, Naar-King S, Wright K (2007). Social support and disclosure as predictors of mental health in HIV-positive youth. AIDS Patient Care & STDs.

[28] Wagner G, Linnemayr S, Kityo C, Mugyenyi P. Factors associated with intention to conceive and its communication to providersamong HIV clients in Uganda. Matern Child Health J. 2012;16(2):510-8. doi:10.1007/s10995-011-0761-5. 10.1007/s10995-011-0761-5 21359828

[29] Cooper D, Harries J, Myer L, Orner P, Bracken H (2007). “life is still going on”: reproductive intentions among HIV-positive women and men in South Africa. Soc Sci Med.

[30] Beyeza-Kashesya J, Kaharuza F, Mirembe F, Neema S, Ekstrom AM (2009). The dilemma of safe sex and having children: challenges facing HIV serodiscordant couples in Uganda. Afr Health Sci.

[31] Kakaire O, Osinde M, Kaye D (2010). Factors that predict fertility desires for people living with HIV infection at a support and treatment centre in Kabale. Uganda Reprod Health.

[32] Kipp W, Heys J, Jhangri GS, Alibhai A, Rubaale T (2011). Impact of antiretroviral therapy on fertility desires among HIV-infected persons in rural Uganda. Reprod Health.

[33] Oladapo TO, Daniel OJ, Odusoga OL, Ayoola-Sotubo O (2005). Fertility desires and intentions of HIV-positive patients at a suburban specialist center. J Natl Med Assoc.

[34] Smith DJ, Mbakwem BC (2007). Life projects and therapeutic itineraries: marriage, fertility, and antiretroviral therapy in Nigeria. AIDS.

[35] Yount KM, Langsten R, Hill K (2000). The effect of gender preference on contraceptive use and fertility in rural Egypt. Stud Fam Plan.

[36] Oosterhoff P, Anh NT, Hanh NT, Yen PN, Wright P, Hardon A (2008). Holding the line: family responses to pregnancy and the desire for a child in the context of HIV in Vietnam. Cult Health Sex.

[37] Laher F, Todd CS, Stibich MA, Phofa R, Behane X, Moha L (2009). A qualitative assessment of decisions affecting contraceptive utilization and fertility intentions among HIV-positive Womenin Soweto. South Africa AIDS Behav.

[38] Heffron R, Davies N, Cooke I, Kaida A, Mergler R, van der Poel S, Cohen CR, Mmeje O (2015). A discussion of key values to inform the design and delivery of services for HIV-affected women and couples attempting pregnancy in resource-constrained settings. J Int AIDS Soc.

